# Causes, Cure, and Prevention of Idiocy

**Published:** 1850-07-01

**Authors:** 


### Art. II.—CAUSES, CURE, AND PREVENTION OF IDIOCY.

This subject is occupying at this moment much public and professional attention. We are rejoiced to be able to say so. The condition of the poor idiot has been sadly overlooked, at least in this country. Psychologists, however, are disposed to make ample amends for their past "sins of omission," and the great question of the curability of idiocy, in all its forms, is now being scientifically tested in more than one portion of the United Kingdom. We have been much pleased with an article on the subject, based upon some American reports of the state of idiocy in that part of the civilized world, which appeared recently in a transatlantic contemporary, of great ability.* The paper in question is based upon some reports which are not easy of access in this country,† and as we agree in the main with the writer's views, we make no apology for transferring his observations to our own pages. The writer justly remarks:

Next to the poverty of the body—nakedness and hunger—the poverty of the mind is occupying public attention and anxiety, and the insane have become, and are becoming, the objects of interest. But it must be confessed, that this interest was excited in former times generally, and in many places even now, not so much for the good of the suffering lunatics as for the security of the healthy people. The insane were supposed to be too dangerous to others to be allowed to run at large; and they were, therefore, confined in strong places, and other means of security were used, so that the timid public should suffer no injury from them. Some were enclosed in worse places than violent criminals; and the jail, both here and elsewhere, divided its accommodations and its discomforts between the law-breakers and the lunatics; for their recovery was not a thing sought for or expected, and no plan was laid, nor means provided, for this purpose. These patients were deemed as permanent nuisances, and were merely to be kept out of harm's way, but out of the way of injuring others.

But, in the course of time, it was discovered that even these hopeless sufferers could be improved; and that most of them, if attended to in proper time and manner, could be restored to health. Consequently, the insane, in many places, are provided with all the means of restoration, and receive as tender and as successful care as those who are afflicted with other diseases.

* The American Journal of Medical Science, edited by Dr. Hays.

† Report made to the House of Representatives of Massachusetts on the Commission of Lunacy. House Doc. No. 72, pp. 10. Boston, 1846.

First Report of the Legislature of Massachusetts by the Commissioners appointed to inquire into the Condition of Idiots within the Commonwealth, by Samuel G. Howe. House Doc. No. 152, pp. 20. 1849.

Second Report of the Legislature of Massachusetts by the Commissioners appointed to inquire into the Condition of Idiots within the Commonwealth, by S. G. Howe. pp. 147. Boston, 1848. Senate Doc.

Causes and Prevention of Idiocy. pp. 24. By S. G. Howe. Boston, 1848.

Notwithstanding these judicious provisions for, and attention to, the wants and sufferings of the lunatic, there are many whose disease sank into dementia, and they are now left in hopeless fatuity.

There was another and much larger class, who were born without mental capacity, or with it, at most, very imperfect; or, in whom it had never been developed. These were the idiots. It could not be said, that they were to be restored to health, because they had never been in any higher condition from which they had fallen. Their state was not a disease, that should be cured; nor a perversion from which they could be drawn back; but it was an original want of power or defect of mental development.

Believing that nature had unalterably fixed them in their present condition, that, as one class of men were created for intelligence and self-direction, so these were created for idiocy and dependence, the world has hardly entertained the suspicion that they could be improved, and has therefore left them to themselves in their degradation, and only provided for their animal wants, and protected them from harm.

Besides these congenital idiots, there were many others who had, in their early years, more or less intelligence, and gave promise of becoming like other men and women; but, from some cause or other, and often from no cause that was known to their families or friends, their minds withered away, and they sank into idiocy as deep and as confirmed as those who were born to this condition.

All these, the demented, the congenital, and the supervened idiots, constitute a large class in every country, and bear very heavily upon every community for their care and support.

That fallacious document, the "United States Census of 1840," states, that there were then 1271 idiots and insane in the State of Massachusetts, and 17,456 in the whole Union.

Certainly there are as many as, and, without doubt, many more than are here stated; for, wherever any trustworthy inquiries have been made, the numbers of the insane and idiots have been found greatly to exceed the statements of the national census.

These idiots were the outcasts of society everywhere, and yet they were dwelling in the bosom of many families. For them and for their friends, for those who supported them, and for the keepers who sometimes guarded them, there seemed no hope of amendment. There was, rather, a fear, and even an expectation, that they would grow worse. The best that they could look for was, that they might go through, and end their low and imperfect life as they had begun it.

But, about fifty years ago, accident suggested to some persons in France, that idiots might be improved. Some philosophers of the sensualists' school, wishing to prove that all our ideas were received through the senses, undertook to teach a wild boy who had been discovered wandering in the forest. He had no language, and apparently no ideas. But, after ineffectual attempts to teach him according to the sensualists' theory, it was discovered that he was an idiot. Yet the labour of Itard, the instructor, was not all lost, for it showed him that his pupil, however low and small his intellectual powers might be, could be educated in some degree. Itard, becoming interested in this matter, continued his efforts in his enviable work, until he was convinced, not only that his single pupil could be improved, but that other idiots could be benefited by education.

Various attempts were made in France to do something for this class, and with a success proportioned to the wisdom and energy of the efforts. Among the most active was Mr. Edward Seguin, who, by his writings and his example, enlisted others to co-operate in the same work. Mons. Ferrus established a school for this purpose in Paris, in 1828, and Dr. Voisin established another in 1839. Dr. Leuret and Mr. Vallée also lent their powerful aid to the work. The result of all these efforts proved, that humanity, even in its feeblest and most degraded condition, can almost invariably receive some advantage from education; and that the dormant power can be roused, and the darkened intellect can receive some light from proper instruction.

In the winter session of 1846, the Legislature of Massachusetts authorized the governor

"To appoint three persons to be commissioners to inquire into the condition of the idiots in this Commonwealth, to ascertain their number, and whether anything can be done for their relief, and to make report of their doings to the next general court."

Happily for the success of this scheme, Dr. Samuel G. Howe, the philanthropic director of the Perkins Institution for the Education of the Blind, was appointed chairman of this committee of inquiry. He laid his plans with good judgment, and pursued them with his accustomed energy.

In the winter of 1847, the commissioners made their first report. But they had made but a partial survey of the State. They had sent circulars of inquiry to the clerks of every town and city. They had personally visited as many towns, and examined as many idiots, as possible. They had obtained information from France, Prussia, and

Switzerland, in regard to the manner and success of the treatment of idiots in the schools which had been established for their education in those countries.

By all these means the commissioners had obtained much valuable information, but not all that was desired. They were, therefore, authorized to continue their inquiries through another year.

At the session of 1848, Dr. Howe laid before the legislature the result of his second year's labour in this work; and the whole is published by order of the government, in a pamphlet of 100 pages of text, and an appendix of tables extending through 48 pages more.

These reports of Dr. Howe contain a great amount of important information relative to the personal and social condition, and the numbers, of idiots, and to the supposed causes of idiocy; and are, therefore, valuable contributions to science.

They treat of the number of idiots in Massachusetts; definition of terms *idiot* and *idiocy;* capacity, condition, and treatment of idiots in private families and in almshouses; cleanliness, alimentation, and exercise of idiots; of European schools for idiots, and of a proposition for the same in Massachusetts. In the supplement Dr. Howe treats of a classification of idiots, the supposed causes of idiocy, parentage, hereditary tendency to bodily and mental imperfection, circumstances which predispose to idiocy, physical and moral condition of parents, intemperance, self-abuse, intermarriage of relatives, and attempts to procure abortion.

The appendix contains several tables, which describe the 574 idiots who were examined, showing their origin, present condition, and future prospects.

Dr. Howe personally visited many towns and examined a great many idiots. Besides this, a competent and trustworthy agent, Mr. Enos Stevens, was employed for the same purpose. In course of the years 1846 and 1847, they visited 182 towns, containing, at the last census, 392,586 inhabitants, and discovered 755 idiots. If the same proportion prevails in the whole state and nation, there are 1418 idiots in Massachusetts, and 32,827 in the United States. They carefully examined 574 of these idiots, and made a record of their names, their physical, moral, and mental character and condition, their parentage, history, and the probable causes of their disability, and the whole, with the exception of their names, is published in the Report before us.

There are many minute details respecting the parentage, the habits and condition of the relations of the idiots, the bodily state and mental power of the subjects, the size and form of their heads, &c., which to a careless observer may seem trivial and irrelevant. But as "this whole subject of idiocy is new, and science has not yet thrown her certain light upon its remote or even its proximate causes, nothing connected with them can be too minute to be observed by the philosopher who is in search of the origin of this low condition of man."—*Second Report.*

Dr. Howe is a believer in phrenology, and has brought the principles of that philosophy to bear upon this subject; and although he has not attempted to base this investigation upon that science, yet he has availed himself of its classification of the powers and qualities of man in the conduct of his inquiry.

It seems to be established by the result, that the mental and moral conditions are connected with the general bodily and the cerebral organization, the stature of the idiots, the size and shape of their heads, and the width and depth of their chests. These have been measured, and the shape of their whole frames and limbs have been examined, and the record of each fact is published.

A large portion—fourteen pages of the Second Report—is taken up with an attempt to define idiocy, or rather an attempt to reconcile the many various and conflicting definitions with which the medical and legal writers have endeavoured to describe this state. Our space will not allow us to enter upon this part of the subject, nor should we hope to be more satisfactory than others who have gone before us. Of all the definitions that we find, none include all who are indubitably idiots, without including some who plainly belong to the more intelligent classes of mankind: and these definitions are so unlike, that no two of them, when applied, would include precisely the same number of individuals.

There is and can be no distinct and definite line drawn through society, on one side of which it can be confidently said that all are idiots, and on the other side all are of sound mind. The several moral and mental qualities and powers that enter into and make up the mind and character of man, are very unequally distributed. One person has much of one faculty, and a disproportionately small quantity of another, and very little of a third, and perhaps none of a fourth. And another may have a very different distribution of power, and be strong in those in which the first is weak, and weak where he is strong. Thus, No. 413 has perception of musical sounds and ability to count, much above the average of men of sound minds, but his skill in the use of his perceptive and reflective faculties is much below them; but No. 139 has a very small arithmetical power, and is dull in regard to musical sounds, while his perceptive faculties are equal to the average of mankind, and his reflective faculties only half as strong. Then we often see a man who is sufficiently wise in ordinary affairs, but has no skill in numbers—or very acute in his perception of facts, but very dull in reasoning from them.

From the lowest idiot, who cannot even control his muscular power so much as to move his limbs or masticate his food, who can neither see, nor hear, nor feel, up to men of the highest order of intellect, there are all intermediate grades of intelligence, without intervals between them. And the mental and moral qualities are distributed in such various proportions in these persons, that it is impossible to classify them strictly.

Idiocy is a defect rather than a disease, a deficiency of the several powers in greater or less degree, rather than a disease of the powers that are originally perfect. Dr. Ray calls idiocy a defective development. This agrees with Esquirol, who says: "Idiocy is not a disease, but a state in which the intellectual faculties have never been manifested." Most of the legal descriptions point to this. Blackstone says, "an idiot, or natural fool, is one who hath no understanding from his nativity." The old English law recognises the same origin.

After quoting several definitions of idiocy, Dr. Howe says, "Without pretending to scientifie accuracy, idiocy may be defined to be that condition of a human being in which, from some morbid cause in the bodily organization, the faculties and sentiments remain dormant or undeveloped, so that the person is incapable of self-guidance, and of approaching that degree of knowledge usual with others of his age."—*Second Report*, p. 19.

There are all degrees of this condition, from the weak-minded man, who errs in judgment and needs counsel of others in the conduct of his affairs, to the lowest idiot, who is but a mere organism.

Writers have made various divisions, each one according to his view of some prominent traits or defects of idiots. To these Dr. Howe adds a division of his own, which is as applicable as any that have been offered, and certainly is as convenient and practicable, inasmuch as it has regard rather to the degree of helplessness and dependence of idiots on others for direction and support:

"IDIOTS of the lowest class are mere organisms, masses of flesh and bone in human shape, in which the brain and nervous system have no command over the system of voluntary muscles; and which consequently are without power of locomotion, without speech, without any manifestation of intellectual or affective faculties.

"FOOLS are a higher class of idiots, in whom the brain and nervous system are so far developed as to give partial command of the voluntary muscles; who have consequently considerable power of locomotion and animal action; partial development of the affective and intellectual faculties, but only the faintest glimmer of reason, and very imperfect speech.

"SIMPLETONS are the highest class of idiots, in whom the harmony between the nervous and muscular systems is nearly perfect; who consequently have normal powers of locomotion and animal action; considerable activity of the perceptive and affective faculties; and reason enough for their simple individual guidance, but not enough for their social relations."—*Second Rep.*, p. 61.

Idiocy, like insanity, may be intellectual or moral, or both, and it may include all or any part of those classes of powers, and in any variety of combination.

Having determined as nearly and stated as clearly as possible what idiocy is, and what idiots are, Dr. Howe next describes the condition of those who had been examined.

Of the 574 idiots, 420 were so from birth, and 154 were originally intelligent, but became idiotic in subsequent years.

Most of them are poor, and a large proportion are public paupers; 22 have property of their own held by guardians; 62 belong to wealthy families; 225 belong to indigent families, but are not public paupers; 220 are town or state paupers; the pecuniary condition of 45 was not ascertained.

In regard to their dependence or power of self-sustenance—

"Fifty-three are as helpless as infants; 74 are as helpless as children two years old; 94, as children seven years old; 138 can work to some small profit, if carefully watched and directed; 179 can nearly earn their board if directed in work by others; and 36 can earn their board and clothing under the management of discreet persons."—*Second Report*, p. 22.

This shows very plainly the absolute and entire dependence of most of this class, and the partial dependence of the rest, on the sound and the healthy for support and direction.

Their ages range from six months to 103 years; eleven are under five; forty-nine under ten; 200 under twenty-five; 372 over twenty-five years of age; and the ages of two are not stated.

The great end of all this inquiry was to ascertain the capacity of idiots for improvement. Dr. Howe thinks that 174 of the congenital idiots and 22 of the supervened idiots under twenty-five years of age, and 195 congenital and 97 supervened idiots over twenty-five years old, are capable of improvement. These are proper subjects of education; they can be taught to do some kinds of labour, to acquire some kinds of knowledge, to attend to their own persons and take care of themselves.

Of the younger class 13 congenital idiots, and of the older class 38 congenital and 38 supervened idiots, appear to be capable of little or no improvement.

Besides their helplessness and dependence, the situation of these idiots is deplorable indeed. Dr. Howe says of the public paupers: "They are of all sorts and grades of idiocy, from the mere simpleton who cannot take care of himself, to the drivelling idiot who wallows in his filth." "Some are comparatively free from the dominion of animal lust and appetite, and are mild, affectionate, and docile; others are a helpless prey to dreadful passions, depraved appetites, and disgusting propensities."

Some want instruction, and, if properly encouraged and directed, will co-operate with a teacher in their education; others are as insensible and unimprovable as the oyster, and can receive no advantage from others, except to be fed, clothed, and sheltered.

A large portion of these idiots are kept in public almshouses, and although Dr. Howe says that he met with no instance of wilfully unkind treatment of idiots by keepers of any almshouses, and that "in most cases the overseers of the poor have given orders for the idiots to be treated with kindness," yet they suffer for want of proper management; for however humane and discreet in ordinary affairs the keepers of these houses may be, yet that special character which is best fitted for the direction of idiots, the peculiar talent which can best understand their degree of intelligence, and adapt its use of motives and its plans of action and government exactly to their comprehension, docility and power, is not sought for; and if it is found in any of the keepers, it is rather accidental than the result of design and care on the part of the public authorities.

In consequence of their disability of mind and body, idiots are incapable of taking so much care of their own persons and doing so much for themselves as others do; they therefore need more care and aid from others to keep their bodies in proper condition. They have a lower sensibility, and their skins are not irritated by foreign matters which may gather upon them; and, moreover, their eyes are not offended by the sight, nor their nostrils by the foul odour, of a filthy surface. They require, therefore, an unusual amount of attention to keep them in a neat condition, and to preserve them from offensive filthiness of person.

But for pauper idiots, generally, the means of this extraordinary attention are not provided; and, according to the report, "in a great many of our almshouses they are disgustingly filthy. They change their body and bed-linen only once a week, and never bathe." In this last matter, idiots form no remarkable exception to a very large part of the people, and we fear that if this were to be adopted as a test of competent mind, very many who are considered as of sound mind, would be thrown into the class of imbeciles.

Idiots do not need more cleanliness than other persons; but they do need as much, and it requires much more care from others to maintain their external purity; and if they are neglected, they become more filthy and offensive.

Idiots have generally great appetites, and many of them eat voraciously. Minute inquiry was made as to the quantity of food which 444 usually ate, and this quantity was compared with that which others of the same sex and age usually eat, and the result shows, that 20 consume less than the average; 81, just the average quantity; 343, more than the ordinary allowance; and 116, just double the amount that others eat, and the average for the whole 444 was about 50 per cent. more than that required for other persons.

Besides this enormous allowance which they obtain by consent of others, they often steal more, and some will devour the offal and the waste of the kitchen, even the foulest and filthiest garbage, which is intended for the swine.

This gluttony increases their natural stupidity, for the nervous energies which might go to sustain a muscular or mental action are all absorbed in sustaining the digestive process.

Idiots are prone to inaction. They do not love motion, and still less, labour. They prefer to bask in the sun, and lie there in utter quiescence of both body and mind. If they are required to work, they do it so unskilfully, and need so much direction and persuasion, that their labour is unprofitable, and those who have the care of them find it easier to support them without, than with, their help. Consequently, very few of them work for the profit or advantage of the labour. Still fewer take any action for the sake of the exercise, and for health. They will not do it voluntarily, and others are unwilling to urge it upon them. Their bodies are therefore sluggish, and their minds stupid. They have weak muscles, and, though their frames may be sufficiently developed, and their limbs sound, yet it is rather from fat than muscular fibre, of which they have comparatively little.

The general management of the idiots in private families is not much better than in almshouses, and in many it is much worse. Of the 354 who were examined in private houses, only 5 were treated very judiciously. These were submitted to the best influences for education and direction; they were taught all that they could learn; their powers were developed to the fullest extent; their propensities and passions were therefore controlled or restrained, and they were made comparatively happy and useful.

But these idiots are generally found in the poorest and most ignorant families: they are the children of the weak and the foolish, and sometimes of other idiots like themselves. There is, therefore, manifested in these families a gross ignorance, both of the causes and of the nature of their disability. Their children are thus subjected to the worst influences, the most improper treatment, and, in some cases, to the strangest experiments for their improvement.

"Sometimes they find that their children seem to comprehend what they hear, but soon forget it: hence, they conclude that the brain is soft, and cannot retain impressions, and then they cover the head with cold poultices of oak bark, in order to tan or harden the fibres. Others, finding it is exceedingly difficult to make any impression on the mind, conclude that the brain is too hard, and they torture the poor child with hot and softening poultices of bread and milk; or they plaster tar over the whole skull, and keep it on for a long time."—*Second Rep.*, p. 32.

Some give mercury to act "as a solder, to close up the supposed crevices in the brain," &c.

Some encourage their children in their ravenous gluttony, because they think the poor imbeciles have no other enjoyment than appetite, and they shall be indulged in that.

In conducting this investigation of idiots, each one was examined personally; and inquiry was made of the friends in regard to every point that would throw any light upon their present condition, or its origin. Their parentage, their health, habits, powers, propensities, were ascertained; their stature, their chest, and the size and shape of their heads were measured. The answer to each inquiry was noted in a memorandum book, and the whole digested and arranged into tables, which we have in the second Report. The names are omitted in the printed table; but each individual is numbered, and against this number are placed the answers to the forty questions that were asked.

The questions related to—1. Age. 2. Commencement of the defect, congenital or not. 3. Height. 4. Temperament. 5. Tactile sensibility. 6. Command of muscular contractility. 7. Dynamic condition of the body. 8. Sensibility to musical sounds. 9. Skill in the use of language. 10. Capacity of fixing the sight on visible objects. 11. Ability to count. 12. Consumption of food. 13. Manifestation of the amative feelings. 14. Depth of chest. 15. Width of chest. 16. Greatest circumference of cranium. 17. Greatest diameter of cranium. 18. Diameter from the root of the nose to the occipital spine. 19. Transverse diameter over the ears. 20. Arc of cranium from the root of the nose to the occipital spine. 21. Arc from ear to ear. 22. Size of the lower frontal region. 23. Skill in the use of the perceptive faculties. 24. Size of the upper frontal region. 25. Skill in the use of the reflective faculties. 26. Size of the lateral region. 27. Activity of the faculties of self-preservation. 28. Size of the posterior region. 29. Activity of the social sentiments. 30. Size of the coronal region. 31. Activity of the moral sentiments. 32. Size of the cerebellum. 33. Activity of the animal nature. 34. Degree of ability to support themselves. 35. Parents in normal condition or not. 36. Parents drunkards or not. 37. Number of cases of idiocy or insanity known among near relations. 38. Scrofulous or not. 39. Given to masturbation or not. 40. Teachable or not. 41. Remarks.

The 1st, 3rd, 14th, 15th, 16th, 17th, 18th, 19th, 20th, 21st, and 37th questions are answered in numbers positively, in regard to each one. The 4th is answered according to the preponderance and order of the nervous, fibrous, sanguine, and lymphatic temperaments. The highest is placed first, and the lowest last, against each idiot's name or number. The 5th, 6th, 7th, 8th, 9th, 10th, 11th, 12th, 13th, 22nd, 23rd, 24th, 25th, 26th, 27th, 28th, 29th, 30th, 31st, 32nd, 33rd, and 34th questions are answered in numbers, relative to the size, power, and development of the same, in 1000 ordinary persons of the same age and sex. In these, 10 is assumed as the normal standard of size, power, &c., and those of the idiots are stated in numbers higher or lower, according to the fact. Thus, No. 395, who ate the average quantity of food, is stated as 10; No. 360, who ate double the average quantity, is stated as 20; No. 259 has no language, and is marked 0; No. 268 can use monosyllables, and is marked 1; and No. 190, who talks as other persons, is marked 10.

It may seem forced to state the sensibility to musical sounds, or activity of the moral sentiments or reflective faculties, in numbers, yet it is the most convenient way of showing the relative quantity or force of the powers or affections. And as the comparison was made on the spot, and at the time when the answers were obtained, it is probable that these numerical statements are correct. The standard, however, is arbitrary, and various persons, who associate with society of more or less cultivation and talent, and have different fields of observation, will have different standards, and consequently different notions of the power of these idiots. Yet we know of no better way of making and stating the comparison, and we put forth these statements of Dr. Howe in confidence that the whole will be sufficiently understood for the purpose of the author, who merely wished to give the best notion of the mental, and moral, and physical condition of these idiots.

The 2nd, 35th, 36th, 38th, 39th, and 40th questions are answered "yes," or "no."

In the last column, there are other remarks in regard to most of the persons examined, mostly in reference to parentage, health, habits, and condition of parents and other relations, and also in reference to the history and habits of the idiot.

This table includes idiots of all ages, from a babe of six months to a superannuated idiot of 103 years. Comparatively few are given in the earliest years. Of the 418 congenital idiots whose ages are stated, only 43 are under ten, giving a proportion of 114 per 1000 of all, in the first decade of life; whereas the whole population of Massachusetts has 237 per 1000 of all, in this decade. Probably many, perhaps most of the idiotic children, may have escaped the notice of the commissioners.

Dr. Howe says that "there are a great number brought into the world so deformed, that it is apparent that they must be idiotic, and so feeble that they do not live through infancy." "Idiots of the lowest class perish in great numbers in infancy and childhood: fools last longer; and simpletons attain to nearly the ordinary longevity. Perhaps it is safe to say, that the average longevity of the lowest class of idiots is not more than six years." And in the opinion of the commissioners, the average duration of all congenital idiots is not more than twelve years.

As we are not sure that we have all the congenital idiotic children, we cannot determine the probable average longevity from this number. The average of all these is 28 years and $2\frac{1}{2}$ months. The average age of all over ten years old is 31 years and $5\frac{1}{2}$ months. That of all the whites of the State of the same age is 31 years and 9 months. So far as any inference can be drawn from this, an idiot, if he survive his tenth year, will live nearly as long as a person of sound mind. This would doubtless be an error. Probably the true longevity of all idiots, and of all above ten years old, is less than those above stated. This viability depends, in great measure, upon the perfectness or imperfectness of their organization. Hence idiots of the lowest class, who have the lowest organization, perish early, while simpletons, whose intelligence and organization are not much lower than those of other persons, and who are saved much of the anxiety, wear and tear of life, that affect responsible men, often live to a great age. The one whose age is now 103 years belongs to this class.

"When a person appeared in infancy, or early childhood, to be idiotic, he is considered to have been born so." Those idiotic persons who never manifested more power than they now appear to possess—who, as they passed from infancy to childhood, did not put forth the talents of the new age—who, however low they may now be, have fallen from no higher degree of intelligence, are considered as congenital idiots. They belong to the class whom Dr. Howe considers as helpless as infants two years old.

There is another class, who appeared in infancy to be as bright as other infants, and even when they were young children they showed no observable deficiency, but, when they passed from this stage of life to the next older, they put forth no more power, and remained ever afterward as weak and helpless as young children of seven years old. These are supposed to have some native defect of organization that prevents any greater expansion of intellect, under the ordinary influences, than they now exhibit.

Dr. Howe says—

"It was probably the intention of the legislature to use the word 'idiot' in the popular and common sense. We have considered, therefore, all persons whose understanding is undeveloped, or developed only in a partial and very feeble degree, or who have lost their understanding, without becoming insane, to be proper subjects for examination."—*Second Report*, p. 21.

Of the 594 who were examined, 420 are reported as congenital idiots, and 154 became idiotic after birth: according to the table, 29 of these in youth, and some in early manhood, were of sound and active mind, and became afterwards insane and then idiotic. The idiocy of the 154 is not caused, so far as can be known, by any defect of organization, but by some external cause or some habits or disease in the course of life.

Here arise a confusion and doubt which, Esquirol says, many fall into in regard to the distinction between idiocy and dementia. Shall those who have once been intelligent, but gradually fell afterwards into the idiotic stupor, without the intervention of lunacy, be considered as idiots or as demented persons? Esquirol solves the difficulty, by disregarding the symptoms, and looking only at the history in making his distinction. "Idiocy is a state in which the intellectual faculties have never been manifested."—*Maladies Mentales*, ii. 284.

"A man in dementia is deprived of that which he has once enjoyed. He is a rich man become poor. The idiot has always been poor and wretched."—*Maladies Mentales,* ii. 285.

*The stature* of 288 idiots was measured. The average height of 172 males was 64·7 inches, and of the 116 females, 60 inches. These facts would be very valuable, if we had the standard height of healthy men and women with which we could compare them. Unfortunately we have no such data. If, however, the general notion, that 67·5 inches is the average height of adult males, this measurement will corroborate our opinion, that idiots have a smaller stature than other persons.

The *width and depth* of the chest were ascertained in 224 adults, and their average corresponds precisely to that of other persons.

*Temperament.*—The temperaments of 417 idiots were ascertained and reported in the table. The four classes—nervous, sanguine, lymphatic, and fibrous—are stated in the order of their preponderance against each of the 417 persons. In 143 the fibrous, in 139 the nervous, in 102 the lymphatic, and in 33 the sanguine, predominated, and stand first in the table.

*Tactile or cutaneous sensibility.*—Some have very great, even an unnatural cutaneous irritability; others have very little sense of touch, so that they are not disturbed by flies and other insects on the skin, and take no pains to brush them off. This is not owing merely to general want of power in the nervous system. One female, case No. 210 in the catalogue, 19 years of age, has so little cutaneous sensibility, that she takes no notice of flies on her skin, and very little of the prick of a pin or pulling her hair. "But she is quite animated by the sound of music, and will leave off eating to listen to it." She is therefore marked 4 as to sensibility of touch, and 13 as to musical sounds, 10 being the standard of each. The average of the 476 examined was 8·52.

*Command of muscular contractility.*—Idiots fail in the command of their muscles, and therefore they cannot control their limbs and direct them with the energy or precision of others. Hence they often walk with a waddling gait, and they make poor mechanics, for want of power to use tools and strike with exactness. The average of the 444 examined was 8·33 to 10 when compared with other persons.

*Dynamic condition of the body* refers to "the general vigour of health as manifested in the ability to put forth muscular strength. 504 were examined to ascertain this, and the average was 7·88 to 10 as compared with other persons of their age."

Probably both the last averages are too large. Dr. H. very properly says, that the former is too high. If a more careful examination could be made of the power and muscular control of these idiots, with tools that require precision of action, and with protracted labour that requires continued effort, a different and lower result would be found in regard to them.

*Sensibility to the musical sounds,* like other powers, is very various: some have none; others have it in a high degree; one is marked 18, almost double the average of men, and there are all grades between them. Yet idiots are generally dull in this respect, and the average of the 300 who were examined was only 6·3, less than two-thirds of the power that is found in the rest of mankind.

*Skill in the use of language,* which is often made the test of intelligence, is generally very small in this class of persons. A few enjoy it as other persons. Of the 452 who were examined, one is marked as high as 13, one 11, and ten are marked 10, and a very few 9 and 8; the rest are lower, and four have no language at all, and are therefore marked 0, and thirty-one are but little better, using only a few monosyllables, and are marked 1. The average of the whole 452 is 5, one-half the skill of the rest of mankind.

*Ability to count* is another test of power which was applied, and by which they were found wanting. Many cannot count at all—they do not see the difference between 2 and 3. Many can count 1 and 2; if we give them one block, and ask how many they have, they may answer one, and then two when we add another; but when we add a third and a fourth, they still do not see more than two. 461 were examined with this view, and five had no conception of numbers, and are marked 0. 157 had some power, though in the lowest degree, and are marked 1. On the opposite extreme, six are marked 10, the usual average. One is marked 13, two 15, one 16, and one 18, having a power in the use of numbers which would be deemed extraordinary, even among those who are sound in mind.

"No. 175 has little use of language. He is marked but 6 in that column; his intellect is very limited; he is, to all intents, an idiot; yet he has an astonishing power of reckoning. Tell him your age, and he will, in a very short time, give you the number of minutes. He is marked 18; he should, perhaps, have been marked higher." The average of the whole 461 is only 3, less than a third of the power of men of good condition.

Some idiots are unable to *fix their eyes upon small objects;* they stare and gaze, but they do not see with distinctness. The image of the object is formed on the retina, but no exactly corresponding sensation is excited in the brain. In this power there is less deficiency than in others; 367 of 442 have the average power of fixing their eyes, and are marked 10; nine have it in an unusual degree, and are marked 11. The average of all is not given, and probably it would not fall much below that of other men.

*Size and form of the head.*—The idiots who were examined have somewhat smaller heads than others, but there is not so great a difference in this respect as is commonly supposed, as will be seen by the following averages of measurements. The first column of figures is the number of idiots who were examined, the second is the average measurement of idiots in inches, and the third is the average size of ordinary persons. M. and F. denote male and female:—

| | | | | |
|---|---|---|---|---|
| Greatest circumference of cranium . . . | M. | 99. | 21·9. | 22·0. |
| Ditto ditto . . . . | F. | 59. | 20·7. | 21·5. |
| Diameter from root of nose to occipital spine . | M. | 94. | 7·5. | 7·8. |
| Ditto ditto . . . . . . | F. | 87. | 7·3. | 7·5. |
| Transversed diameter over the ears . . . | M. | 94. | 5·5. | 5·8. |
| Ditto ditto . . . . . . | F. | 87. | 5·3. | 5·5. |
| Arc of cranium from root of nose to occipital spine | M. | 87. | 13·3. | 13·8. |
| Ditto ditto . . . . . . | F. | 61. | 13·0. | 13·5. |
| Arc from opening of one ear over to opening of the other . . . . . . . | M. | 89. | 14·0. | 14·3. |
| Ditto ditto . . . . . . | F. | 61. | 13·5, | 14·0. |

Yet there is a very great difference in regard to the manifestation of the several powers and propensities that are supposed by phrenologists to be connected with several parts of the head.

Dr. Howe was the president of the Phrenological Society, and is skilled in the practical application of phrenological principles. The American editor observes—"His agent also was accustomed to cranial measurements, to craniological examinations, and to observing the connexion between the cerebral developments and the mental and moral manifestations. Phrenology has therefore every advantage of skill, practice, and faith in its truth, to establish itself more firmly by means of this investigation, and to give further proof of the soundness of its doctrines, in the correspondence between the prominence or deficiency of certain parts of the cranium, and the strength or weakness of certain powers, sentiments, or propensities."

But the following table, containing the result of Dr. Howe's observation, shows, at least, that he has found no new proof of practical phrenology in this new field of inquiry.

*Comparison of the development of certain parts of the cranium, and of the mental and moral manifestations, with the same in* 1000 *ordinary persons, those in the class of sound persons being assumed as* 10 :—

| | |
|---|---|
| Development of the lower frontal region of the cranium . . . | 9 |
| Skill in the use of the perceptive faculties . . . . . . | 5 |
| Development of the upper frontal region of the cranium . . . | 9 |
| Skill in the use of the reflective or reasoning faculties . . . | 3 |
| Development of the lateral region of the cranium. . . . . | 8 |
| Activity of the faculties of self-preservation, as cautiousness, cunning, &c. | 4 |
| Development of the posterior region of the cranium . . . . | 8 |
| Activity of the social nature, or attachment to others . . . | 6 |
| Development of the coronal region of the cranium . . . . | 9 |
| Activity of the moral sentiments . . . . . . . . . | 6 |
| Development of the region of the cerebellum . . . . . . | 7 |
| Activity of the amative feelings . . . . . . . . . | 14 |
| Average activity of the animal nature, estimated by the developments of amative feelings, the dynamic condition of body, and the consumption of food.* . . . . . . . . | 10 |

For ten of these statements, 116—and in the eleventh, 114—in the twelfth, 70—and in the thirteenth, 115 idiots were measured and examined, and these are the results, and they are generally corroborated by the measurements and examinations of most of the others.

The average development of the several parts of the cranium of these idiots is, in comparison with that of sound persons, as forty-three to fifty—that is, nearly as large. But the average power of the moral and mental faculties supposed to be connected with them, as compared with the same faculties in ordinary persons, is as twenty-four to fifty, less than one-half.

But, on the contrary, the cerebellum, which has a much smaller proportionate development, as seven to ten, is connected with moral manifestations which are all equal to, and some much larger than, the same in men whose cerebellum is of the ordinary size. The activity of the amative feelings is as fourteen to ten, and the activity of the whole animal nature in these idiots is just equal to that of others, or as ten to ten.

This great deficiency of power, in one case, may just be what a phrenologist would expect from the small deficiency of cerebral development. "We are not sufficiently acquainted," says the reviewer, "with the minutiæ of this science to determine what gradations of power should accompany certain gradations of development of the brain. Nor are we prepared to say, whether the excess of the

* Second Report, Appendix, p. 52.

amative feelings ought not to be expected from the diminished cerebellum. We make no deductions from these facts. This we leave to those who wish to establish or disprove the phrenological system from them. We have no desire to do either. We will only say, that these facts come to us from unquestionable authority, whose principles and habits would lead him to give due attention and weight to all facts that can have any bearing upon this science, but whose impartiality and regard to truth impel him to state every fact precisely as he finds it, whatever may be the conclusion to which it may lead."

Dr. Howe says,—"It may be stated here, in general terms, that the result of this examination and measurement shows, that no dimensions of the head, except extreme diminutiveness, and no shape whatever, can be relied on as criteria of idiocy. A few of the worst cases of idiocy are those in which the head is normal as to size and shape. Nevertheless, the tables show, that, taking the aggregate of all the cases, an obvious relation is seen between the size and development of the cranium, and of its different parts, and the amount of intellectual power and of the different kinds of mental manifestation." —*Second Rep.*, p. 65.

*An inordinate appetite* ranks high among the propensities of the idiot's animal nature. More than a quarter of those examined consumed double the usual quantity, and some were insatiable with any amount of food. The average of the whole is fifteen to ten, as compared with the food of other persons.

In some, the excessive eating was enormous. One child of five years was in the habit of taking a gallon of milk daily, and "one boy of thirteen has been known to drink six quarts of water a day," and another, after being reduced and limited as to his food, now consumes just double the average used by others.

*The manifestation of the amative feelings* is the saddest part of this whole picture; it reveals oftentimes the cause, and sometimes the terrible consequences, of idiocy. In seventy idiots who were examined, this propensity has a power, compared with that in sound persons, as fourteen to ten. "In some cases it amounts to perfect mania," and it continues long after the period of youth, even into old age. One person, sixty-six years old, is given now to open and shameless masturbation. Another of sixty-three is given to "excessive venery," another of sixty-five has been masturbating for forty-five years, because, in these cases, although "the physical power breaks down, the dreadful propensity continues unabated." Among idiots masturbation is a very common vice; out of 389 idiots who were examined, 204, of whom seventy-five are females, are known to practise it frequently. Cases were discovered of even little children addicted to this destructive habit; and, worse than this, nineteen little children were countenanced in this practice by intemperate, foolish, or degraded parents or nurses, and even some took satisfaction in this evidence of their children's precocity.

*Physical condition.*—Idiocy is often connected with other defects. Of the 574 idiots, 21 are blind, or have deformed eyes, 12 are deaf, 23 have deformity of mouth and nose, 54 have deformed hands or feet, 96 are paralyzed in some parts, 14 are torpid in feeling, 125 are subject to fits; convulsions are produced in three by use of tobacco, and in 29 by anger; faintness, nausea, and vomiting are produced in 7 by fright. 491 out of 497 who were examined are scrofulous.

*Degree of ability to support themselves.*—In the long table of individual idiots, in which is stated in numbers the degree of comparative ability of each one to support himself or herself, 13 are said to be able to do nothing, 48 can do one-tenth, 75 two-tenths, 96 three-tenths, 138 four-tenths, 175 five-tenths, and 32 six-tenths of the amount of labour sufficient for their support. And the average ability of the whole is a little more than one-third sufficient for this purpose.

There is some discrepancy between this statement and that which we quoted before, in which it was stated 220 are as helpless as children two and seven years old. But probably this might be explained by the author. Perhaps this tabular and numerical statement refers to the possible condition and power of the idiots when under proper guidance, and the former statement to their present condition and power, unaided by others. Nevertheless, both go to establish the same principle, the dependence of this class of persons on others for their support.

There is another element to be included in this estimate—that is, these persons can do as much as is herein stated, "under the management of discreet persons." Alone, their earnings would be much less, and probably in most cases nothing; and when under the care of indiscreet persons, as are the parents of most of them, they do little or nothing towards their own maintenance.

*Pecuniary circumstances.*—These idiots are mostly the children of poverty. Of the 529 whose pecuniary condition was ascertained, only 22 had property of their own, and 62 belonged to wealthy families, 225 are members of poor families, and 220 are public paupers.

*Age.*—200 of these are under, and 374 over 25 years of age.

Dr. Howe thinks favourably of their *capacity of improvement.*

He says "that 196—almost the whole of the younger class—and 272—more than three-fourths of the older class—are capable of being improved in some degree, and raised somewhat from their present miserable condition."

*The causes of idiocy* are matters of the greatest interest. Why are so many, why are any human beings found in this low, wretched, and dependent condition? This is a question that ought to interest the physiologist, the philanthropist, and the political economist. The causes of this low degradation of humanity should be ferreted out, and, if possible, removed, and the increase or the future production of idiots prevented.

Not knowing what are the necessary causes of this condition, we must be content with learning what are the preceding or co-existing events or circumstances, that, by their general or universal precedence to, or connexion with, idiocy, may be presumed to stand as causes. In this matter, Dr. Howe has done a good work. Without pretending to settle this question beyond all doubt, he has thrown much and valuable light upon it. He inquired into the history and condition of each case. He ascertained the character and health of the parents, the early and subsequent health of the subject, his or her organization and propensities, his or her habits, exposures, and indulgences; and the result of each inquiry is stated in the table of his report.

The causes which produce idiocy—which prevent the development of the ordinary mental and physical powers that are essential to normal life, or impair or extinguish them after they have become partially or entirely developed—are probably many and various.

The first thing to be observed is the "low condition of the physical organization of one or both parents."

It is not our intention on this occasion to give a dissertation on hereditary character, or the transmission of qualities from parent to child. This subject requires more time and space than we now can give to it. Yet we hope ere long to see it discussed in all its bearings here or elsewhere, and the connexion of generations explained so clearly, that the world may be warned and put on its guard against every habit or action that may prevent the perfectness of health in the children.

We would merely say in passing, that we believe that parents can give to their children no other qualities or powers than those which they themselves possess, and that, whatever may be the condition of either parent when the germ, or the element, or the pabulum of life is given to their offspring, that condition, or the peculiarity of organization which is susceptible of that condition, will probably be transmitted to the child.

Dr. Howe has great faith in this principle, and rests much of his argument and explanations upon it; and he has shown so many facts to corroborate it, that it is not easy to withhold our confidence in his reasonings and conclusions. He says, "if ever the race is to be relieved of the tithe of bodily ills which flesh is now heir to, it must be by a clear understanding of, and a willing obedience to, the law which makes parents the blessing or the curse of the children; the givers of strength, and vigour, and beauty, or the dispensers of debility, and disease, and deformity."

We have already shown, that very few of these idiots, whose history was ascertained, were the children of healthy parents. If they inherited any constitution from their progenitors, it was a feeble one at least; and, in many instances, there was a positive tendency to disease or weakness. Many idiots are the children of idiots or simpletons. Some whole families of these are idiotic, and others are mixed, being in part idiots and in part simple or weak-minded, and others are sound.

Fifty idiots were discovered, whose parents were one or both idiotic or insane.

Forty-five parents have each two idiotic children; thirteen have three each; eight have five each; one has five; one has seven; one has nine; and one has eleven.

The report does not say whether, in any or all of these seventy families, there were other and sound children; nor whether, in these cases, both or only one of the parents were insane or idiotic. Another remarkable fact shows the hereditary tendency. "In fifteen families, all of the children of the first marriage were idiotic or puny, while all those born of a second marriage of the surviving healthy parent with a healthy person were sound in body and mind."

The idiotic taint or hereditary tendency may be considered as the remote or predisposing cause, which in many persons lies dormant, until it is stimulated to action by other and proximate or exciting causes, and then both together produce idiocy. Or the hereditary taint, acting alone, may cause only weakness, which under the best influences and education may be counteracted, and thus the child may prove to be merely a weak-minded man; or under other influences, the child may grow to be a simpleton, or have some oddities in his character. But under bad influences, such as the care or neglect of weak, foolish, or wicked parents or associates, the child may become an idiot. In the same family, there may be various exciting causes acting on the different children, and producing a corresponding variety of character among them. One child may be merely weak, another simple, a third odd, and a fourth idiotic.

Some of the children of tainted families receive the predisposition to idiocy from their parents, and carry it with them through life; but very careful management and judicious education avert all exciting causes, and these persons pass respectably through the world—yet they may transmit their hereditary taint to their children. Then this third generation, if not as well trained and guarded as their parents, may meet with the exciting causes and become idiotic. Or, if they pursue the faithful course of their fathers, the taint may still lie dormant in them, and they may escape, but yet possibly transmit the taint to the fourth generation, who may or may not be idiots, according to their education and self-management. In this way we may explain the apparent irregularity of hereditary character, and the re-appearance of idiocy, insanity, or other hereditary disease in the third or fourth generation, after the second or third has enjoyed a perfect immunity from it.

Idiocy, therefore, although a hereditary disability, does not affect every successive generation, nor all the collateral branches of the same generation. Hence we find idiocy scattered among the various individuals or branches of the same family, touching some and omitting others. In such families, forty-nine idiots had one near relative idiotic; nine had two; six had three; four had four; six had five; three had ten; and one had nineteen, near relations like themselves.

The report does not state how many idiots were examined for this purpose, or whether any, or how many, of the other 496 idiots were ascertained to have no relatives like themselves. We are therefore unable to make any deductions from this statement, as to the proportion of idiocy which may seem to be hereditary, or the proportion which may be entirely original in the subject.

Very few idiots marry. This is a blessing, and so far it is a safeguard to the race. Humanity requires that the succession of idiots should be arrested. Yet many weak-minded persons within a few shades of idiocy marry, and leave another generation more weak or simple than themselves. Some persons who have been temporarily insane or demented marry, and send their taint, or their liability, or one or the other of these conditions, down to their children.

But the most lamentable and certain, though less frequent, cause of congenital idiocy is the lasciviousness of some female idiots, whose illegitimate offspring are almost always, like themselves, idiotic and lustful.

Some persons who are irrepressibly addicted to masturbation are advised to marry, as a means of protection from this ungovernable propensity. This, probably, is very well for them—it may occasionally answer the intended purpose, but it is a cruel thing for their children. It entails upon them, perhaps the same propensity, certainly a feeble constitution; often weak minds, and sometimes idiocy. Twelve of these idiots are the children of parents who were thus married. The other children of the same families, if there be any, if not idiotic, are probably feeble in body and mind, and enjoy a lower degree of life than the children of better or more healthy parents.

*The near relationship by blood* of the parents seems to be the cause of, or, at least, it is the precedent fact to, many cases of idiocy. We do not suppose that this connexion is, of itself, the cause of idiocy. But if there are any weaknesses, or defects of body or mind, or tendencies to disease, or oddities, in the family, they may be overpowered, or cease to appear, in the next generation, if those who have them marry with strangers, and mix their blood and life with those who have not these peculiarities: and thus the children may escape the imperfections or liabilities that otherwise might have been entailed upon them. But when two persons of the same blood and character unite together in marriage, their peculiarities are doubled in power by being combined in their children; and the odd or weak traits, which were subordinate in the parents, may predominate in their offspring.

In the course of this inquiry the parentage of 359 idiots was ascertained. In seventeen families the parents were near blood-relations. In one of these families there were five idiotic children born; in five, four each; in three, three each; in two, two each; and in six, one each. In these seventeen families ninety-five children were born: forty-four idiots, twelve scrofulous and puny, one deaf, and one a dwarf; fifty-eight in all of low health or imperfect, and only thirty-seven of even tolerable health.

The parents of Nos. 59, 60, 250, 251, were cousins, and had, besides these four idiot children, four that were deformed.

*Intemperance of parents.*—The habits of the parents of 300 of the idiots were learned, and 145 (nearly one half) are reported as "known, to be habitual drunkards." Such parents transmit a weak and a lax constitution to their children, who are, consequently, "deficient in bodily and vital energy, and predisposed, by their very organization, to have cravings for alcoholic stimulants." Many of these children are feeble, and live irregularly. Having a lower vitality, they feel the want of some stimulation. If they pursue the course of their fathers, which they have more temptation to follow and less power to avoid than the children of the temperate, they add to their hereditary weakness, and increase the tendency to idiocy in their constitution; and this they leave to their children after them. The parents of case No. 62 were drunkards, and had seven idiotic children.

Seven of the congenital idiots were the children of prostitutes; seven others were illegitimate.

The *condition of the mothers during gestation* has some influence upon the health and character of the offspring. The commissioners made inquiry as to this matter in regard to as many as possible, and discovered that two of the mothers of the idiots were insane, two were drunken, sixteen were sickly and feeble, and seven of these last suffered from fright, one had fits, and received a blow on the abdomen, during their pregnancies, and another suffered from violent parturition. All the children who were born from these gestations were idiots from birth, with the exception of two, whose mothers were sickly, and one whose mother was injured. These three became idiots afterward.

*Attempts to procure abortion* may be injurious to the child, even though it be carried through, and safely delivered at the end of the full period. At least seven children were made idiots, says the report, by these unsuccessful attempts. Young women thus sometimes try to get rid of their burden, or conceal their shame; not succeeding, they afterwards marry, and the child is born at the proper time, but is idiotic. Other children are successively born of the same parents, and give no evidence of ill health or unsoundness of mind. Several cases of this kind are among those alluded to. One woman had seven sound children, and another had six, born in wedlock, though the oldest child of each of them, upon whom abortion was attempted, was idiotic.—*Second Report*, p. 90.

Looking upon idiocy rather as a deficiency of power, or as a disability, than as a disease—as a negative, rather than as a positive condition—it is easy to suppose that it may be produced by a single cause, or by the co-operation of several causes which would not individually be sufficient to produce this condition, yet each may contribute its portion of influence to produce this deterioration of mental and physical power, and aid in making the child an idiot. Case No. 89 is an idiot of the lowest kind: he cannot walk, or hardly creep; he cannot feed himself with a spoon; nor can he speak. He has, apparently, no intellect. His father was intemperate, and was nearly related to his wife by blood. Her family were tainted with idiocy, as she had an idiotic cousin. She was much terrified and distressed in mind during the early part of her pregnancy, and was sick, and carried her child with much difficulty through the latter part, and, "finally, her confinement was very long, protracted, and painful." Possibly, any one of these circumstances—the intemperance, hereditary taint, intermarriage of relatives, the fright, illness, or difficult parturition—occurring alone, would not have produced idiocy in this case, for the same parents had other children that were not idiotic; yet any one of these may have the effect very materially to diminish what would otherwise have been the bodily and mental vigour of the child, to lower his tone of life, and carry him so far toward idiocy: and thus the added or combined effect of all these depressing causes may be sufficient to produce the idiocy that was manifested in the offspring.

If, as we have supposed, the parents can give to their offspring no other constitution than that which they possess at the time when they impart their life to the child, then the low organization, the ill health, the folly, the wickedness of the parents, or whatever reduces their power of body or mind below the normal standard, must prepare the way for a still greater deterioration or lower degradation in their children. If, then, these hereditary weaknesses in the children are not overcome by proper training, or if their hereditary tendencies are not resisted and counteracted by the force of proper education and management, by the cultivation of the highest health, and the avoidance of every depressing cause acting on life, and of every exciting cause of disease or idiocy, these children will transmit to the next generation a lower degree of physical and mental vitality.

These causes existing in the parents produce idiocy in the children, or that feeble and imperfect organization upon which, when other exciting causes may be added, idiocy may supervene.

Besides the hereditary taint or depression of constitution, there are many personal causes which operate directly upon the subject, and produce original idiocy in him.

*Masturbation* appears to be the most prominent among these depressing causes. The habits of 389 idiots were examined in regard to this matter; and 204, more than half, were found to be addicted to it. And, what is still more worthy of notice, several children—two of four years, one of seven, two of eight, five of nine, one of ten, two of eleven, three of twelve, and one of thirteen, (seventeen, not yet fourteen years old!) were addicted to this disgusting and exhausting habit. No. 447 is stated to have been "healthy and intelligent until taught masturbation at *six!*" No. 343 is only nine years old, and has been addicted to "masturbation *many* years!"

In some this habit is irrepressible, and in some it is "openly and shamelessly indulged." It does not always cease with youth, but is, in many, continued far beyond the middle age; eight idiots between 50 and 60, nine between 60 and 70, one 78, and one 80 years old, are reported as still addicted to this vice.

The venereal appetite, either from original organization, or from frequent provocation or indulgence, is very strong in idiots. Besides the many who are reported as masturbating, or as open prostitutes, fifteen are reported as "very lustful," or given to "excessive venery;" and even four idiots, who are more than 60 years old, and one nearly 70, indulge in this vice. One of 63 is given to "shameless venery," and another of 64 is "still lustful to excess."

*Intemperance and fits* are also prominent among the supposed exciting causes of supervened idiocy.

These habits and conditions of the parents and progenitors of idiots, and of the idiots themselves, are thus stated, not as the entire and unquestioned causes of their present low state, but as the probable causes, and such as are supposed to be so by the families or acquaintances of the idiots.

Besides the several items, which are arranged in forty columns, and registered against each individual, as before stated, there are also some other remarks, which could not be so easily classified. We are unable to give any farther analysis of these, and yet we offer some quotations as specimens.

"No. 57. Supposed cause, violence during parturition, sickly gestation; subject to fits till 14.

58. Supposed cause, drunkenness of mother in gestation.

59. }

60. } Parents related, and subject to insanity. Of 8 children, 4 are idiots, and 4 deformed.

61. Parents intemperate.

62. Parents related; had 16 scrofulous children, 3 of them idiots.

63. Brother to 403; parents drunkards.

64. Supposed cause, sickly gestation.

65. Father drunkard, and mother scrofulous.

66. Parents scrofulous and sickly."

"162. Deformed, gluttonous, and pachydermatous.
163. From masturbation and gluttony.
164. Insane and intemperate at 20 years of age.
165. Full of sores, and always puny; skin pachydermatous.
166. Salivated in infancy; effects still continued.
167. Mother had fits during gestation, and received a blow upon abdomen.

No. 168. Mother and grandmother scrofulous; nephew to above.
169. Fits in childhood, and formerly a drunkard.
170. Mother a simpleton. He is given to masturbation and venery.
171. Scrofulous and deformed; growth of bones arrested early.
172. } The parents of these were simpletons, cousins, and drunkards; have
173. } 4 children foolish."

" 339. Father drunkard; and the race all scrofulous.
340. Mother insane; cousin idiotic.
341. } Brothers: only children of a scrofulous mother and a drunken
342. } father; masturbation and fits from 10 years old.
343. Father intemperate; masturbation many years.
344. } Masturbation; very scrofulous breed.
345. } Brother of the above.
346. Very scrofulous breed.
347. Gluttonous; parents intemperate; mother a prostitute; sister a simpleton.
348. Very scrofulous mother; father has healthy children by another wife.
349. Father drunkard; full of scrofulous sores."

These show from what a low and degraded race most of the idiots have sprung, and what is their wretched condition now. Idiocy is thus so generally connected with, or produced by, the depressing causes acting on the health and life, with the exhausting habits, circumstances, or exposures that have nearly or remotely gone before it, or immediately co-exist with it, that it may be considered as merely the last step in vital depression.

In the long line of humanity, there are infinite numbers of degrees, from the highest, where is perfect health of body and mind, to the lowest, where is idiocy. Whatever wasting habit, circumstance, or exposure, such as intemperance, debauchery, gluttony, or other depressing cause, acts upon a person standing in any of these degrees, exhausts some of his vital power, and carries him downward, more or less, toward idiocy, and he is thereafter a lower man, weak-minded or simple, or foolish, or idiotic, according to the force and protraction of the depressing cause or causes.

It is to be regretted, that this commission could not have extended its inquiries through the whole State, and revealed the full extent of the misery and degradation that have thus fallen upon humanity. But this investigation was established only for a definite purpose, which was, to ascertain the number and condition of the idiots, and also, whether these could be improved, and whether enough of them were teachable to justify the establishment of a school for them by the State. The government justly inferred, that the 574 idiots, who were examined, and whose condition and character were learned, were sufficient to indicate the character of the whole, and therefore discontinued the commission, and proceeded to provide the means of educating them.

Low and disheartening as is the picture which we have here given of the mental and physical condition of idiots, it is not without hope. Dr. Howe thinks that almost all of them—488 out of 574—are capable of improvement, to a greater or less extent.

Two strong inducements for public action or interference to relieve or diminish idiocy now present themselves. 1st. The enormous expense of supporting 1400 persons—about one-five-hundredth part of the whole population of this commonwealth—in a state of idiocy: and 2nd. The motives of humanity, to give, if possible, to these wretched creatures some idea of responsible life, some means and power of self-sustenance, and some self-respect.

There are two modes of action pointed out. One attempts to remedy or mitigate the evil by educating such of the idiots as can be provided with the means; the other strikes at the root of the future idiocy, and endeavours to remove the causes and prevent the recurrence of the disability hereafter. But we must confess, with great pain, that weighty as is the task to do the first, greater and more hopeless is the last. It is far easier to teach these stupid idiots, even to create intellect where it does not seem to exist, than to reform the morals of men and women, whose habits or indulgences lead to idiocy in themselves, or in their children, or to impress upon the world the necessity of looking only to the interests of the next and future generations in their marriage-contracts, and in the management of their own persons.

The extinction of idiocy must be a work of ages. Nevertheless, it can be accomplished in the course of time. The causes that weaken or corrupt the human constitution, and produce ill-health or tendency to idiocy, or idiocy itself, may be removed. The successive generations of the weak, the unhealthy, and the tainted, may be each improved, and raised, and strengthened in some degree. By carefully educating the children of the feeble and corrupted families, by guarding them against the errors of their parents, by teaching them to avoid the exciting causes of idiocy, the hereditary taint may be kept dormant, and even diminished, until finally, through the course of successive generations, it shall be extinguished, and hereditary idiocy appear no more.

For the education of idiots there is encouragement to hope. The experiments which have been made in France, Switzerland, and Prussia, prove that many of these, who otherwise would be idiots of a low order, may, by proper training, be raised to such a condition, that they may live in and enjoy the comforts of their families, and that others may be made self-dependent, and pass respectably and happily through life.

This, however, requires a peculiar kind of training. The usual influences of home, and especially of the ordinary homes of idiots, and the teaching and discipline of common schools, are not sufficient for the education of this class of persons; they must have schools, teachers, and apparatus, peculiarly adapted to their capacities and powers.

We believe that there were, until lately, no such schools in America, and that all the idiots of this continent were left to grope their way in their original darkness and degradation, except a few rare cases, whose intelligent parents provided the extraordinary and proper means for their education.

The legislature of Massachusetts, at its last session, after receiving these reports from Dr. Howe, appropriated the sum of twenty-five hundred dollars annually, for three years, to educate ten idiots, in order to try the experiment; and the whole was placed under the general direction of Dr. Howe. Mr. James B. Richards was selected as a teacher, and went to Europe to visit the schools which are there in progress, and to learn their method of training and educating idiots. Mr. R. returned in September last, and in October opened his school at Boston with four idiots, and has now nine.

Although nothing decisive as to the extent of the power or the capacity of idiots can be inferred from this small experiment, and the short time during which these few have been under discipline and instruction, yet enough has been done to show, that even these stupid and apparently unimpressible children can be roused, and taught, and influenced.

To form any proper notion of their progress, it is necessary to know their condition when they came to school. One boy of fourteen was brought from a poor-house, where he had always worn women's clothes. He is now dressed as other boys are, and enjoys his new garments, and uses them as properly as they do. He could not go up or down two steps, without getting upon his hands and knees; now he walks up and down, though with some hesitancy, in the common manner. Five of these boys had no control of their urinal or alvine evacuations; now they control them during the day, and mostly during the night. They could not be trusted to feed themselves, and some would steal food from the kitchen, or elsewhere, and one would devour the offal that was set aside for the swine. Now, they all eat as other boys, under the supervision of their teachers, who determine the quantity of their food. One who had no use of his feet can now walk with assistance. Within the first month, one boy of nine years learned to throw wood from the ground on a pile, and another made still farther progress, and learned to pile it straight, laying the sticks parallel with each other. They learned to climb a ladder on the under side with their hands and feet, and when they reach the top, they can turn around one of the rails to the other side, and return by the upper surface. They could not walk on either side of the ladder when they entered the school in October.

They had no conception of numbers, or of the size or form of objects. Now some of them can count as far as *six* or *seven;* they understand the difference between a square and a round object, and will select a quart or a peck, or other measures, when asked to do so.

In their whole appearance and manner, there is more of self-respect, and intelligence, and activity; and one cannot fail to observe the very great difference in the expression of their countenance, when comparing their daguerreotype likenesses, which were taken when they entered, with their faces as they now appear.

It will not be suspected that we make these statements as proofs of great success or progress, but only to show that the beginning of the experiment offers sufficient encouragement for perseverance; that the idiot's mind is not entirely blank; and that he is not, like the brutes, immovably fixed in present low rank, and irrecoverably doomed to remain there, in darkness and degradation, for ever.

---

